# Research evidence use in local government-led public health interventions: a systematic review

**DOI:** 10.1186/s12961-023-01009-2

**Published:** 2023-07-03

**Authors:** Jennifer L. Dam, Phoebe Nagorka-Smith, Alex Waddell, Annemarie Wright, Joannette J. Bos, Peter Bragge

**Affiliations:** 1 Monash Sustainable Development Institute, Monash University, 8 Scenic Boulevard, Clayton Campus, Clayton, VIC 3800 Australia; 2 School of Health and Social Development, Institute for Health Transformation, Deakin University, 1 Gheringhap Street, Geelong, VIC 3220 Australia; 3 Action Lab, Monash University, 8 Scenic Boulevard, Clayton Campus, Clayton, VIC 3800 Australia; 4 Victorian Department of Health and Human Services, 50 Lonsdale Street, Melbourne, VIC 3000 Australia; 5 Melbourne School of Population and Global Health, The University of Melbourne, 207 Bouverie Street, VIC 3053 Carlton, Australia

**Keywords:** Local government, Research evidence use, Public health policy

## Abstract

**Background:**

Local governments play an important role in improving public health outcomes globally, critical to this work is applying the best-available research evidence. Despite considerable exploration of research use in knowledge translation literature, how research is practically applied by local governments remains poorly understood. This systematic review examined research evidence use in local government-led public health interventions. It focused on how research was used and the type of intervention being actioned.

**Methods:**

Quantitative and qualitative literature published between 2000 and 2020 was searched for studies that described research evidence use by local governments in public health interventions. Studies reporting interventions developed outside of local government, including knowledge translation interventions, were excluded. Studies were categorised by intervention type and their level of description of research evidence use (where ‘level 1’ was the highest and ‘level 3’ was the lowest level of detail).

**Findings:**

The search identified 5922 articles for screening. A final 34 studies across ten countries were included. Experiences of research use varied across different types of interventions. However, common themes emerged including the demand for localised research evidence, the legitimising role of research in framing public health issues, and the need for integration of different evidence sources.

**Conclusions:**

Differences in how research was used were observed across different local government public health interventions. Knowledge translation interventions aiming to increase research use in local government settings should consider known barriers and facilitators and consider contextual factors associated with different localities and interventions.

**Supplementary Information:**

The online version contains supplementary material available at 10.1186/s12961-023-01009-2.

## Introduction

According to the World Health Organisation (WHO), non-communicable diseases (NCDs) such as heart disease, respiratory disease, cancer and diabetes are the leading causes of chronic illness and premature death globally [1]. Research on the social determinants of health—the conditions, forces and systems that shape the conditions of daily life [1] highlights that NCDs are largely influenced by modifiable behaviours (or risk factors) such as tobacco use, physical activity and diet, and that these behaviours are often shaped by the local environments people live in; underscoring the important of role of local governments in improving population health outcomes [2].

Often articulated as the level of government closest to the people [3], local governments are the metropolitan and regional areas that sit within a state, territory or province. Their connection to a defined population and place means they are well positioned to influence health outcomes through both ‘bottom-up’ engagement with local stakeholders and ‘top-down’ policy interventions [3]. For example, local governments provide social infrastructure such as community health services and sporting facilities which play a critical role in addressing the challenge of ‘lifestyle’ diseases and improving health outcomes [4, 5].

Decentralisation of public health responsibilities has seen the role of local government expand considerably in many regions [6–8]. While this is broadly understood to benefit health outcomes, bringing decision-making within closer proximity of service delivery [3], it has not been without its challenges, including a lack of resources and the need to navigate new decision-making structures and processes [7, 8]. Alongside this, local governments have also faced growing demands for greater use of research in health policy [9]. In Australia for example, local government public health responsibilities in the state of Victoria are mandated through the [10] which is underpinned by several principles, including “*evidence-based decision-making*” (s. 5) to guide the effective use of resources and inform public health interventions.

An evidence-based approach in public health—broadly defined as the integration of the best available research with community preferences and other resources such as practitioner expertise—is associated with both improvements in health outcomes and enhanced organisational efficiency and service delivery [11]. Within local government settings, an evidence-based approach requires policymakers and practitioners to draw on various forms of evidence including population health data, community feedback, guidelines and research. These forms of evidence need to be weighed against community needs, constituent preferences, strategic imperatives, and availability of resources and expertise [12, 13]. Ideally, this results in interventions with demonstrated impact that are also feasible and acceptable to the community that they seek to serve.

Despite broad consensus on the benefits research in public health policy, it is often underutilised for a range of reasons such as inadequate access to relevant research or a lack of institutional support [14, 15]. This ‘evidence-policy gap’ is a well-established phenomenon across multiple sectors and settings including and beyond local government. Furthermore, research has shown that perceptions of research needs can differ greatly between researchers and policy-makers [16]. Addressing this mismatch is a key goal of knowledge translation (KT) research, which aims to increase the relevance and effectiveness of research-based evidence alongside building individual and organisational capacity to use it [16]. However, after more than two decades of KT research, there remains a gap in empirical studies identifying which strategies are most effective at increasing research use, and how to implement them [17]. Critique of this literature argues that KT studies have tended to focus too heavily on how to get more research *into* policy, emphasising a need to better explore how research and policy interact outside the context of an implementation intervention.

There are a number of related systematic reviews that have examined research use in public health policy [18–26]. These have largely focused on exploring barriers and facilitators of research use [18–20], and influences on research use [21, 22], including the political dimensions of public health policy [23]. More recently, Verboom and Baumann [24] comprehensively mapped various characteristics of qualitative literature describing research use, including geography, methodology and use of theory [24]. While these reviews were predominantly global, and all but one [20] focused exclusively on public health policy, they included policy settings at all levels of government, examining local government experiences alongside national and state or regional government collectively.

Two reviews concentrated on local government settings, however they also included studies relating to local agencies/practitioners outside of local government [25, 26]. One was global with a focus on high income/OECD countries only [25], the other was limited to England-based studies [26]. Although different in scope, both reviews were interested in research use in public health decision-making, drawing attention to the diverse ‘landscape’ of local public health decision-making structures and processes. These reviews emphasise a need for research to explore these differences in order to foster a deeper understanding of the “broad patterns of evidence use (and need)” [26] (p. 9) within local government settings.

Given the increasing expectation for local governments globally to develop and deliver public health interventions, this systematic review aimed to identify, appraise and synthesise published literature describing research use in local government-led public health interventions. Specifically, the review focused on exploring how research evidence was used in local government; for what type of activity (e.g. public policy development, health education, partnership); and variations in evidence use by intervention type.

## Methods

### Design

A systematic review methodology was used and reported in accordance with the PRISMA checklist (see Additional file 1) [27]. Prior to conducting the review, a protocol was registered at Open Science Framework https://osf.io/s38qf/.

For the purpose of this review, ‘research’ was defined as primary evidence produced through formal research or scientific methods, generally based in universities or with university-affiliated researchers, and/or published in peer-reviewed journals [28]. The use of raw data such as health monitoring or surveillance data not generated by academics was not a focus of this review. ‘Public health intervention’ is defined as an action “*intended to promote or protect health or prevent ill health in communities or populations*” [28], including environmental interventions aimed at improving health conditions.

To support the study aim of exploring whether research use varied depending on the nature of an intervention, a public health classification was used to categorise studies according to the type of intervention they described [29, 30]. The use of organising frameworks in research and practice is also understood to support greater consistency in reporting public health interventions, and help facilitate national and international comparison [29]. Developed for the National Public Health Partnership in Australia, the classification used was informed by public health experts and an analysis of core public health functions and existing classification systems in Australia and internationally [29]. It comprises six top-level classes (see Additional file 2):

- functions (e.g. promote health and prevent disease; ensure public health capability);
- health issues (e.g. health and wellbeing; diseases and conditions);
- determinants of health (e.g. environmental; socio-economic);
- intervention methods that support the achievement of public health functions including actions, activities, programs and services;
- the settings in which public health work is undertaken (e.g. local government; education; healthcare); and
- the resources and infrastructure that support this work.

This review drew on the ‘Methods’ sub-classes which encompass a range of methods specific to public health (e.g. health education, community development, Health Impact Assessment), and those that reflect the day-to-day work of public health workers [e.g. administration, management and policy development [29]].

### Search strategy

The search strategy was developed in consultation with a library specialist using key terms based on review objectives and identified through early research scoping. These were adapted as required for each database (see Table 1 for MEDLINE example). The CINAHL, PsycINFO, MEDLINE, Scopus, Cochrane and Health Systems Evidence databases were systematically searched for English language articles published between 2000 and 2020. This time frame was chosen because the year 2000 marks the origins of scientific enquiry of evidence use in public health [11] and the beginnings of legislative requirements for local government public health planning (e.g. Canada, Australia, England, The Netherlands) [31–34]. Additional screening included reference lists of systematic reviews and cited references of studies that reported primary evidence and/or made public health policy recommendations.

**Table 1:** MEDLINE search strategy

Search strings	Search terms
Evidence use string:	‘Evidence use’ OR ‘evidence based’ OR ‘evidence informed’ OR research OR scientific OR EBDM OR EBP OR EBPH OR EBHP OR EIDM OR EIP OR EIPH OR EIHP
Intervention string:	‘Public health’ OR ‘population health’ OR ‘community health’ OR ‘health promotion’ OR ‘health policy’
Setting string:	‘Local government’ OR ‘local authority’ OR council OR shire OR LG* OR ‘city government’ OR ‘county government’ OR ‘government, city’ OR ‘government, county’ OR ‘government, local’ OR ‘government, metropolitan’ OR ‘government, municipal’ OR ‘metropolitan government’ OR ‘municipal government’

### Eligibility

This review included primary qualitative and quantitative peer-reviewed journal publications. To be eligible for inclusion, studies had to report the use of research (including research sourced from grey literature such as policy briefs, agency reports or guidelines) in an intervention aimed at improving human health outcomes at the population level. This could include an intervention targeting a specific risk factor (e.g. tobacco use) or broader factors that influence population health (e.g. social determinants, health equity, environmental health).

In order to generate a picture of ‘everyday’ research use in local policy settings, the review focused exclusively on studies describing research use in public health interventions implemented in local government settings, by local government decision-makers. Studies describing interventions implemented by non-public health departments within a specified local government were included, providing improved health outcomes was an explicitly stated goal. However, studies that reported interventions implemented by public health departments not embedded in a policy setting, or within local government settings by non-public health decision-makers (e.g. university-based research teams), were ineligible. Observational studies examining the role of research in decision-making were included however, KT studies with a primary objective of increasing research use were excluded. See Additional file 3 for a full list of inclusion and exclusion criteria.

### Study selection

Study screening and selection was conducted using Covidence systematic review software [35]. After duplicates were removed, articles were independently screened by title and abstract by two authors (JD and PN) and in full text by three authors (JD, PN and AW). At both stages of the screening process, conflicts were resolved collaboratively, with a fourth author (PB) contributing where consensus could not be reached.

### Methodological quality appraisal

Study quality and risk of bias was assessed using the Critical Review Forms for Quantitative Studies [36] and Qualitative Studies [37] as appropriate to the study design. The Critical Review Forms incorporate both dichotomous (yes/no) and descriptive items to appraise study variables such as methodological rigour, appropriateness of measures and sampling procedures. The Mixed Methods Appraisal Tool (MMAT) [38] was used for studies employing mixed research methods. The MMAT includes two screening questions and five categories of questions to select from based on study design. The response format for all questions is categorical (yes/no/can’t tell). These tools were chosen as they are published, freely available, widely utilised in health sciences and suitable for assessing a range of research designs. The tools enabled quantitative analysis of strengths and limitations within and between included studies. Tallying of categorical variables was used to classify included studies as ‘low’, ‘medium’ and ‘high’ quality. Quality appraisal was undertaken by one author (JD) with input from a second author (AW). A selection of studies (10%) were first appraised by both authors and results were compared and differences discussed until an agreed conclusion was reached to adopt a consistent approach. See Additional file 4 for an overview of quality appraisal.

### Data extraction

Following screening, descriptive data for included studies was extracted and tabulated, including: citation; publication year; whether it was co-authored by local government; study aim/objectives; use of theory; research methods; research setting; participants; intervention type; and the level of detail provided in describing evidence use (see Additional file 5). Data extraction was primarily undertaken by one author (JD) with review from a second author (PN). First, both authors completed a proportion (10%) to allow for comparison of results. Consistency was observed between both authors.

Studies were categorised as follows according to their level of description of evidence use:

- Level 3: the included study made a statement about research use.
- Level 2: level 3 + the study discussed how and/or why research was used.
- Level 1: level 2 + the study described stakeholder experiences of using research and/or barriers and facilitators of research use.

Studies were also categorised according to their intervention focus using a public health classification [29, 30]. For parsimony, studies that reported more than one intervention method (e.g. health education and capacity building) were categorised according to the primary method identified. For observational studies that did not identify a specific intervention (e.g. those exploring research evidence use across broad aspects of public health decision-making), the cross-cutting category of ‘public policy development’ was used.

Following categorisation, further data extraction was undertaken (by JD in consultation with PB) to capture descriptions of research use, including perceived barriers and facilitators. A descriptive analysis of research use was conducted with greater attention given to higher-quality studies that provided more detailed descriptions of research use (i.e. levels 1–2).

## Results

**Fig. 1:**
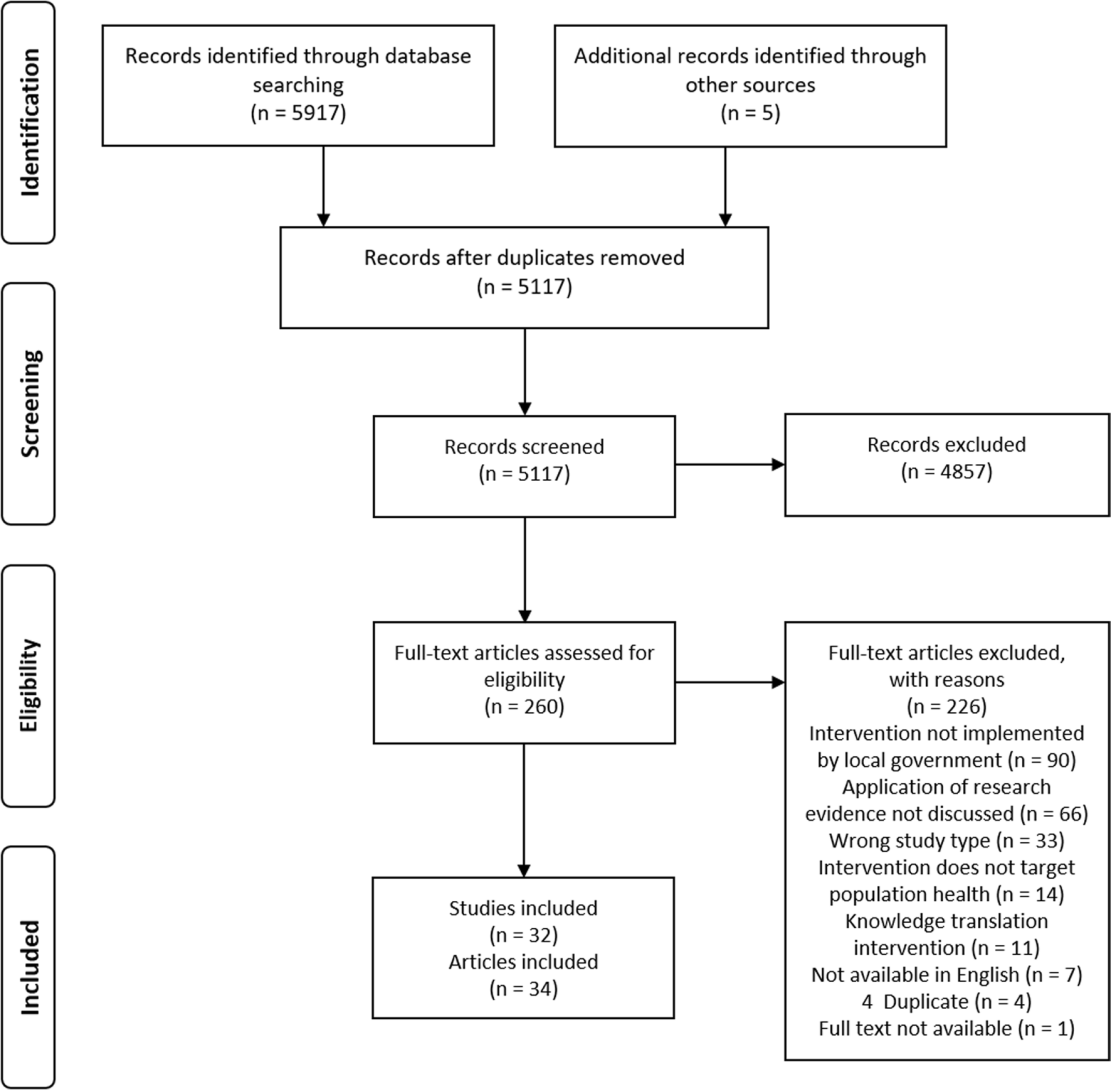
PRISMA flow diagram

Of the 5922 articles identified through searching, 805 duplicates were removed. A further 4857 that did not meet inclusion criteria were excluded through title and abstract screening. The remaining 260 articles were reviewed in full text, and a further 226 were removed (see Fig. 1 for exclusion reasons). A final 34 articles were considered to meet the inclusion criteria [39–72]. Four of these (two sets of two) related to the same study: Atkins et al. [40] and Kelly et al. [41] and; Hunter et al. [44] and Marks et al. [45]. These were analysed together, resulting in 32 included studies (see Table 2). Six studies were included, but not quality-appraised. One did not have a clearly defined research question [66], and five were commentaries [58, 65, 67, 68, 72]. All six described research use in local government-led public health interventions, were authored or co-authored by local government, published in peer-review journals and met all other inclusion criteria. It was determined to include them in the review as they contained relevant data; however, they should be interpreted in this context.

**Table 2:** Study characteristics

First author (year)	Study setting	Research design	Theoretical framework	Study quality (high/med/low)	Intervention method	Public health issue
Experiences of evidence use (level 1)						
Armstrong (2014)	Australia: Victoria	Mixed: Qualitative interviews, Quantitative survey	Evidence-Informed Policy and Practice Pathway; Diffusion of Innovations Theory	High	Public policy development	Services, systems and policies
Atkins (2017) and	UK: England	Qualitative: Interviews	COM-B Model	High	Public policy development	Services, systems and policies
Kelly (2017)						
Beenstock (2014)	UK: England	Qualitative: Thematic content analysis	Realist Viewpoint	High	Public policy development	Services, systems and policies
Hunter (2016) and	UK: England	Qualitative: Interviews, workshops	Kingdon's (1995) Multiple Streams Framework	High	Public policy development	Services, systems and policies
Marks (2015)						
Kneale (2019)	UK: England	Qualitative: Interviews		High	Public policy development	Services, systems and policies
Larsen (2012)	Denmark: National	Quantitative: Survey		High	Public policy development	Services, systems and policies
McGill (2015)^a^	International: England, Brazil, USA and Canada	Qualitative: Focus groups		High	Public policy development	Social determinants/health equity
South (2020)	UK: Yorkshire and Humber Regions, England	Qualitative: Interviews		High	Public policy development	Services, systems and policies
Willmott (2015)	UK: England	Qualitative: Interviews		High	Public policy development	Services, systems and policies
Rossow (2015)	Norway	Qualitative: Content Analysis	Advocacy Coalition Framework/Weiss Conceptual Model	High	Advocacy and lobbying	Alcohol use behaviours
Marko (2020)	Australia: Metropolitan Melbourne, Victoria	Qualitative: Interviews		High	Public policy development	Gambling behaviours
Erwin (2019)	USA: National	Quantitative: Cross-sectional survey		High	Other methods (partnership)	Services, systems and policies
Frew (2020)	UK: England	Qualitative: Interviews, observation		Med	public policy development	Services, systems and policies
Martineau (2013)	UK: England and Wales	Qualitative: Document review, informal discussions		Med	Legislation and regulation	Alcohol use behaviours
Phillips (2015)	UK: England	Qualitative: Observation, interviews		Med	Public policy development	Social determinants/health equity
Purtle (2018)	USA: Philadelphia, Pennsylvania	Qualitative: Interviews, document review		Med	Legislation and regulation	Eating behaviours
Corburn (2007)^a^	USA: San Francisco	Qualitative: Observation, interviews, document review, media analysis		Low	HIA	Social determinants/health equity
Van Vliet (2018)^a^	Sweden: Norrkoping Municipality	Commentary		–	Public policy development	Social determinants/health equity
How/why evidence was used (level 2)						
Gavens (2019)	UK: England	Qualitative: Interviews, focus groups	Critical Realist	High	Public policy development	Alcohol use behaviours
Reynolds (2018)^a^	UK: Greater London, England	Mixed: Ethnographic observation, interviews, surveys		High	Legislation and regulation	Alcohol use behaviours
Boyce (2018)^a^	USA: South Bronx, New York City	Quantitative: Pre-post intervention participant surveys		Med	Health education	Sexual health behaviours
Corburn (2014)*	USA: City of Richmond	Qualitative: observation, interviews, document review (HiAP)		Med	Public policy development	Social determinants/health equity
Von Heimburg (2017)^a^	Norway: Levanger and Verdal	Qualitative: Case study (HiAP)		Med	Public policy development	Social determinants/health equity
Kogel (2020)^a^	Spain: Sant Andreu	Qualitative: Various		Low	HIA	Social determinants/health equity
Elbers (2019)^a^	UK: Leeds City Council	Commentary		–	Research and evaluation	Gambling behaviours
Linzalone 2017)*	Italy: Municipality of Arezzo	Mixed: Focus groups, interviews, surveys		–	HIA	Social determinants/health equity
Rube (2014)^a^	USA: New York City	Commentary		–	Other methods (infrastructure development)	Built environment
Steer (2018)^a^	Canada: Region of Peel	Commentary		–	Public policy development	Tobacco use behaviours
Stated evidence use (level 3)						
Browne (2017)	Australia: Victoria	Quantitative: Frequency counts		High	Public policy development	Services, systems and policies
Dobbinson (2020)	Australia: Brimbank City Council, Victoria	Quantitative: Case–control		High	Other methods (infrastructure development)	Lifestyle behaviours
Dannefer (2020)^a^	USA: New York City	Qualitative: Observation, interviews		Med	Other methods (infrastructure development)	Social determinants/health equity
Lederer (2014)^a^	USA: New York City	Commentary		–	Public policy development	Eating behaviours

Stated research use level 3 = the study made a statement about research use; level 2 = level 3 + the study discussed how and/or why research was used; level 1 = level 2 + the study described stakeholder experiences of using research and/or barriers and facilitators of research use

^a^Denotes studies co-authored by local government

### Study quality assessment and confidence in the evidence

Confidence in the methodological rigour of included studies was good, with mostly high (*n* = 16) and medium (*n* = 8) quality scores (refer Table 2 for quality scores). Only two studies were considered low quality. Overall, studies were clear in articulating their purpose and informing the need for the stated research. Research methods were well described and appropriate for addressing stated research questions. Few studies (*n* = 8) specified the overall research design and many were lacking in describing the role of the researcher and measures to control potential bias. Trustworthiness of included studies was mixed. Common concerns included a lack of detail describing the research site and participant and auditability of data collection and/or analysis procedures. Refer Additional file 4 for an overview of quality appraisal and study level data.

### Study characteristics

Characteristics of included studies are shown in Table 2. Studies were published between 2007 and 2020. More than half (*n* = 18) were published between 2017 and 2020. Studies were mostly qualitative (*n* = 19). A smaller number were either quantitative (*n* = 5) or mixed methods (*n* = 3). Geographically, studies spanned 10 different countries, although a large proportion (*n* = 12) focused on the United Kingdom (UK). Studies were typically undertaken within a specific local government area or a subset of local governments within a specified region. Only one study spanned multiple regions, although they did not explore between-country differences.

### Types of evidence used and associated definitions

Eleven studies reported a specific aim of investigating evidence use, mostly to explore research use alongside other types of evidence (e.g. evaluation reports and community views). Four focused explicitly on use of research, including evidence-based decision-making [43], systematic reviews [51], and evidence-based guidelines [40, 41, 58].

Only four studies included a definition for research evidence [42, 47, 58, 69]. Consistent with the definition guiding this review, all defined ‘research as evidence derived from scientific methods and analysis. Definitions also emphasised the role of non-research-based evidence in public health decision-making such as evaluation reports or data (i.e. to inform the scale of health issues).

### Use of theoretical frameworks

The use of theory as part of the study design was limited in the included studies. Only six studies used a theoretical framework (refer Table 2); three to guide data analysis [40–42, 59] and three more robustly to inform overall research design [39, 44, 45, 50].

The use of theory by local governments to inform intervention development or implementation, as described by the included studies, was even less evident. While some studies described the use of known models or frameworks by government to help inform understanding of particular health issues (e.g. place-based approaches to address health equity), more detailed accounts of theory to guide intervention design, implementation or evaluation were not observed.

### Intervention methods and public health focus

As shown in Table 3, studies described a range of public health interventions. For example, ‘legislation and regulation’ included the development or enactment of local laws and regulations such as licensing requirements and taxes. Interventions targeted a variety of health-related concerns; some were specific (e.g. tobacco use or problem gambling); others focused on broader factors such as health systems or the social determinants of health.

**Table 3:** Summary of intervention methods and public health issues

	*N* = 32^a^	%
Intervention methods		
Advocacy and lobbying	1	3
Health education	1	3
Health impact assessment (HIA)	3	9
Legislation and regulation	3	9
Public policy development	19	59
Research and evaluation	1	3
Other methods (infrastructure development)	3	9
Other methods (partnership)	1	3
Public health issue		
Alcohol use behaviours	4	13
Built environment	1	3
Eating behaviours	2	6
Gambling behaviours	2	6
Lifestyle behaviours	1	3
Services, systems and policies	11	34
Sexual behaviours	1	3
Social determinants of health/healthy equity	9	28
Tobacco use behaviours	1	3

^a^Based on number of included studies not articles

A large proportion of studies (59%) described research use in ‘public policy development’. While some were specific, for example describing public policy development to address alcohol use behaviours [59], many (*n* = 13) adopted a broader focus, describing public health decision-making in general terms in relation to service delivery, planning or strategy development (see Table 3 for individual study details).

### Descriptions of research evidence use

More than half of included studies (*n* = 19) provided detailed descriptions of research use (categorised as level 1). These studies were predominantly medium to high quality (see Table 2). Ten were less detailed (level 2) but did describe how or why research was used. Overall, the quality of these studies was mixed; only two were high quality. Four studies only included a statement about research use (level 3), of which two were high quality. Although less descriptive in their reporting of research use, these studies were more explicit about sources, including identifying primary research that informed interventions.

Experiences of research use were typically framed in terms of barriers and facilitators, which were broadly consistent across different types of interventions. Common barriers to evidence use reported by the studies included:

- lack of consensus about what constitutes research evidence [39, 44–47, 49, 55, 56, 58];
- availability of resources to support research use (e.g. staff skills, time and organisational support) [39–41, 47];
- perceived gaps in the evidence base on key public health issues [51, 65]; and
- the political nature (and associated complexity) of the decision-making context [40, 41, 44–47, 51, 52, 54, 55, 58].

As well as different conceptualisations of what research-based evidence is, perceptions about what it means to be evidence-based also varied [39–42, 47, 55]. Differences were primarily attributed to variations in professional backgrounds of staff, including within local government public health teams (e.g. architecture, physiotherapy, nursing), which had implications for how notions of research, and what is considered *robust* research, were conceived [39, 47, 55].

Several studies described the impact of political influence on research use and the tensions that arose when evidence-based decision-making or public health priorities were in conflict with other political goals and decision-making processes [40, 41, 51, 54]. For example, in the case of local alcohol policy-making where public health priorities conflicted with commercial priorities [50]. While at times political influence was reported to outweigh even good research [44, 45], when political goals were aligned with public health priorities or research findings, this facilitated its use [52]. Other facilitators included:

- individual and organisational capacity to use research [39, 42, 47];
- research findings communicated in clear and simple language [50, 51, 53]; and
- collaboration, including formal partnerships [43, 47, 48, 50, 51, 54–57, 59, 61–66, 72].

The reciprocal benefits of collaboration in knowledge building and sharing were well described. For example, informal networking between local governments was identified as an important research dissemination method; associated with additional benefits such as facilitating greater ‘*buy-in’* and promoting more robust policy responses [59], and reducing duplication of effort by promoting the effective use of limited resources [65]. Participatory processes at the heart of well-established evidence-based methods such as ‘Health Impact Assessment’ and Health in All Policies were also reported to foster research use through facilitated stakeholder engagement (including local citizens and experts from various sectors) across a range of public health issues [57, 62, 64, 66].

Three themes emerged relating to research use. First, was the commonly expressed desire for more *‘localised research’*. Local evidence, including “*evidence of effectiveness in other LGAs*”, was described as critical to informing “*policies and strategies that were most likely to work in their local communities*” [48] (p.373). Although local evidence often referred to evidence that might not constitute research as defined in this review (e.g. evidence derived from community consultation by local governments), a lack of locally relevant research (as opposed to national or international research) was a commonly cited concern. This was described in relation to the use of national guidelines in a number of ‘public policy development’ studies which described a lack of local utility due to their broad focus. For example, a participant in one study noted, “*they lacked specificity and did not take into account complexity and scale*” [40] (p.5), while others felt that the diversity of local populations (and associated public health needs) were not always aligned with national populations and priorities. Consequently, local evidence was not only given precedence over national guidelines, it was considered essential for giving context to public health issues.

In ‘legislation and regulation’ interventions, the need for *‘localised research’* was more specific. For example, in alcohol licensing processes in the UK, Martineau et al. [54] describe how only certain types of evidence could be used. In the case of health-related research, it was only permitted if it was “*legally relevant as well as scientifically valid*” (p. 439); directly linked to licensing objectives (e.g. public safety); “*legally framed in terms of non-health objectives*” (p. 436) and; specific to the geography of the named premise. This study articulated a need for locally situated research linking known alcohol-related harms with local alcohol consumption practices, to facilitate its applicability in licensing processes. While only one study described a ‘research and evaluation’ intervention (reporting the commissioning of local research), it highlighted a range of positive outcomes [65]. In addition to clarifying the extent and nature of the public health issue (i.e. problem gambling), undertaking local research helped to foster partnerships and drive coordinated local and regional action [65].

The second theme to emerge was how research was used to ‘*frame or legitimise*’ public health issues or different points of view [48, 50, 52, 56, 57], particularly when engaging stakeholders outside of public health teams (e.g., other local government departments or community groups). This was observed in ‘Health Impact Assessment’ and Health in All Policies interventions as well as various ‘public policy development’ interventions where research was used to help build awareness of the public health implications of non-health issues; contributing to the adoption of more equitable policies. For example, Marko et al. [48], describe using research to highlight *“the impact of EGMs* [electronic gambling machines] *on broader health and social issues (such as housing instability and family violence)”* (p. 371) to reframe problem gambling from an addiction context to a public health context. However, the legitimising role of research did not always serve to benefit public health outcomes. Analysing research use in ‘advocacy and lobbying’, Rossow et al. [50] observed the use of research by two opposing coalitions to legitimise different points of view; also noting the active undermining, or de-legitimising of public health research by one coalition [50].

The third theme related to the need to ‘*integrate research’* with other sources of evidence, and the work associated with this. For example, in a study exploring research use by local governments in Victoria, Australia, participants reported that a mixture of evidence was considered both “*most useful*” and “*most influential*” in public health decision-making [39] (p. 7). While this finding was similar in numerous other studies, the drivers varied somewhat depending on the nature of the intervention, the stakeholders involved and the breadth or relevance of available research. For example, in ‘Health Impact Assessment’ interventions grounded in evidence-based decision-making, it was reported that gaps in the research-base meant that processes had to rely on other inputs including expert opinions and anecdotal evidence. At other times, presenting research within broader narratives (including local and anecdotal evidence), was seen as important to help influence decision-makers [39, 46, 52]. Most commonly in planning and strategy development activities described in ‘public policy development’ interventions; where stakeholders were often negotiating competing demands, personal and professional differences and power dynamics. While research was also considered important in these decisions, participants reported that it was rarely enough to support the full breadth and complexity of decision-making needs, often due to a lack of local relevance or failure to address certain considerations such as economic impact [49, 52]. Despite the work involved in integrating various forms of evidence, it was clear that it was beneficial in terms of helping to engage different stakeholders (often with competing interests) on different public health issues [55], as well as addressing “*different views about relevant evidence methodologies*” [42] (p. 466).

These themes were often described within broader narratives of decentralisation of public health responsibilities and the associated push for more evidence-based policy. This was particularly prominent in studies from the UK where there was an underlying assumption that local governments were expected “*to up their game and get used to the processes and practices of evidence-based public health*” [40] (p. 9). Responding to this, Atkins et al. [40] argue that research needs to be fit for purpose and consider decision-maker needs, also calling for more shared responsibility in addressing the evidence-policy gap. Differences in evidence needs between local governments and national health services were also highlighted, including the need for a focus on sources rather than hierarchies of evidence.

## Discussion

This is the first known systematic review with an explicit focus on an in-depth exploration of research use in local government-led public health interventions, aiming to identify how research is used, and whether use varies depending on the nature of the intervention. This review found that local governments employ a range of different intervention strategies to address public health outcomes; highlighting the multi-dimensional nature of their role in public health. Furthermore, this review found that how research is used can vary depending on the nature of an intervention and the public health issue being addressed. These findings build on previous KT literature that emphasises the complexity of research use in public health policy, articulating the importance of acknowledging intervention methods and the nature of public health issues alongside the myriad factors surrounding the accessibility, legitimacy and practical value of various forms of evidence in local policy settings.

### Strengths and limitations

A key strength of this review is the use of robust, established methods, including: a comprehensive search strategy (six electronic databases); having two authors independently screen all studies to reduce potential bias in study selection; and independent review of over 10% of studies to help mitigate any potential for bias in data extraction, quality appraisal and classification of interventions. Another major strength of this review is its focus on how research is used; building on findings of related work to examine research use at the level of intervention type and public health issue being addressed. This enabled a more nuanced understanding of *how* research use can vary according to these characteristics. In doing so, this review highlights that steps taken to improve research use in local government may need to vary according to these differences.

There were also a number of limitations to this review including that it was limited to English language articles which may have resulted in the exclusion of some studies. While database searching included the Cochrane review, a more comprehensive search of grey literature was not undertaken, which may mean that some studies were overlooked. This review adopted broad inclusion criteria, which may have resulted in some overlap with studies in previous reviews, however, the exclusive focus on local government settings allowed for findings to be explored in-depth within a singular government setting. Similarly, this review only included studies that described research use in local government-led interventions. While, this was purposeful decision, aimed at capturing a realistic view of research use by local government stakeholders, it should be noted that public health work in local government settings is rarely undertaken by a single agency. Partnership is both an integral aspect of addressing the challenges of public health, and a known facilitator of research use [73]. Although it is expected that this has resulted in the exclusion of studies that involved local government participation in public health interventions, it was considered necessary in order to meaningfully address a known gap in the literature and help build knowledge about local governments use of research. Despite this, an important limitation of this study is the ability to generalise findings across diverse local government settings. As discussed in the introduction, local governments have a globally recognised role to play in public health [4], however, public health is often conceptualised and organised differently both within and across regions [3, 4, 29]. Local government’s capacity to address public health outcomes is highly context dependent and impacted by relationships to higher levels of government, degree of decision-making authority and allocation of budgets and resources [3, 4]. All of which can have a considerable impact on the type of decisions being made, the stakeholders involved and the role of research in decision-making.

### Geography

Despite its global focus, this review had a high concentration of studies from the UK and fewer than previous reviews with an international focus; none were from low and middle-income countries. These differences are likely attributed to the review's exclusion criteria, particularly the exclusion of KT interventions to support the aim of capturing ‘everyday’ research use and the exclusive focus on public health departments embedded in local policy settings.

### Use of theory

Consistent with previous literature [23, 24], the use of theory was limited by both researchers and the governments they were studying. This is notable given the known benefits of theory in facilitating the success and sustainability of interventions and ensuring their replicability in other settings [74, 75]. For example, behavioural theory can help inform the social and cultural dimensions of health behaviours (e.g. smoking) and assist with identifying strategies to promote change [75]. However, with a plethora of theoretical approaches to choose from, theory selection can prove challenging [76]. Future research may consider further exploration of local government stakeholders' knowledge of, and use of theory to inform public health intervention strategies.

### Barriers and facilitators

As with previous reviews [18–20], a large number of studies in this review described research use in terms of barriers and facilitators to use (e.g. 39,42,43,46,53), despite this not being the focus of this review or the included studies. This finding is consistent with a recent review by Verboom et al. (2020) and suggests a shift in focus away from perceived barriers and facilitators in favour of exploring how research is engaged with in policy settings.

### Exploration of research use

Despite a considered focus on exploring *how* research is used, this review found that few studies provided process-orientated descriptions of research use. How research is used was explored across three themes (i.e. the desire for more local research; the legitimising role of research and; the need to integrate research with other types of evidence). While themes were broadly consistent with wider KT literature, by categorising studies according to intervention type, this review identified several unique insights.

### Demand for localised research

Direct interaction with the local environment is intrinsic to the work of local governments [77, 78], and the desire to be locally informed is often in competition with the desire to be evidence-based. Consistent with Kneale et al. [26], this study observed a clear desire for more localised research. This was driven by a range of factors across different studies including: accountability to local constituents [45]; political ideology [40, 45]; beliefs about the uniqueness of local populations and associated health needs [40, 41] and; gaps in empirical literature [51, 64]. It also found that in many cases, these factors led decision-makers to rely on other sources of evidence, often at the expense of methodological rigour or evidence hierarchies [46, 49].

Also highlighting the importance of local research at the local government level, was the requirement for health research to be locally specific to be eligible for use in alcohol licensing processes in ‘legislation and regulation’ interventions as described by Martineau et al., [54]. Although less explored in the literature, this has been observed in other aspects of local government decision-making [25].

Despite critique from study participants about the limited utility of nationally informed evidence-based guidelines in local settings [40, 41], they were still a commonly reported go-to-resource in the face of challenges surrounding the use of primary research (e.g. time and budget constraints) [39, 51]. This is consistent with previous literature [22, 78] and highlights an opportunity for higher levels of government and non-state actors such as researchers, knowledge brokers and peak bodies, to work more closely with local governments to explore how research needs can be more directly addressed through research synthesis and guidelines.

### Elevating local experiences

Although there was a clear desire for more local research, this review found only one example of locally commissioned research [65]. This study provided a detailed, process orientated description of the various stages of the project to meaningfully inform other local governments on local evidence building. As well as underscoring a need for greater investment in production of local research, the relevance of this study to the scope of the present review highlights the potential value in greater inclusion of grey literature and non-traditional research papers in future systematic reviews to help elevate local government experiences.

This may also help address concerns about the tendency for KT literature to be descriptive or theoretical [17] and provide greater insight into what may or may not help to optimise research use. Promisingly, this review, along with a previous review [24], found that the number of studies using observational methods is on the rise; as is local government participation in study authorship compared to earlier literature [20]. This is important as observational studies, such as ethnographies and case studies that give voice to first hand-accounts of local government experiences can provide much needed practical insights into decision-making process and research use [20, 23, 24]. However, if this research gap is to be addressed, studies will also need to adopt a more open-minded approach to ensure greater exploration of policy-making activities and processes, as opposed to identifying perceived deficits in research use [79].

### Framing and legitimising

The use of research to *frame or legitimise* different points of view in policy settings is often explored through the lens of Weiss’s [80] typology of research use. Commonly referred to as symbolic or political use, this involves using research to justify an action or position [81]; as observed in this review in ‘advocacy and lobbying’ [50]. While this type of use is often characterised negatively, Weiss et al. [82] argue it can also be functional; as long as research findings are not distorted or omitted in the process. This strategic use of research by health teams (observed in this review by studies describing the use of research to frame the impacts of non-health related decisions) [48, 56, 62], highlights the persuasiveness of research with some stakeholder groups, and the role it can play in helping to legitimise public health concerns. These findings also underscore the need for unbiased evidence reviews that present a full picture of the various impacts of different health issues and associated interventions.

### Integrating evidence

The importance of drawing on a variety of evidence sources to inform local public health policy is well articulated in the literature [11–13, 77]. This review identified that the drivers for this can vary across different types of interventions, emphasising the importance of building a research-base (including research synthesis) that “*better reflect*(s) *the complexity of local populations and systems of influence in order for this evidence to be more useful and usable in local public health decision-making*” [46] (p. 10). This means addressing demands for more locally relevant and issue specific research, using accessible language and open access publishing, and fostering greater involvement from policy-makers in research production [51].

## Conclusion

This review builds on previous knowledge about barriers and facilitators to research use in public health decision-making, identifying considerable diversity in how research is used, by whom and for what purpose. Consistent with previous literature, this review highlighted the complexity of using research in local government settings, including the associated demands of needing to integrate research with other evidence sources to facilitate its use. In response to these challenges, local government stakeholders expressed a desire for more relevant research that reflects local experiences, supports the implementation of interventions within local communities, addresses the social determinants of health, and is communicated in clear and straightforward language that facilitates engagement with diverse stakeholders. This review classified studies according to intervention type, however other factors associated with local public health policy (e.g. policy cycle) are also likely to shape when and how research is used and as such are worthy of consideration in designing future studies. Additionally, future research should pursue more observational approaches to build further knowledge of how research (including theory) is applied, as well as fostering greater involvement of local government stakeholders in communicating findings. Building on the approach used in this review, researchers may need to adopt a more nuanced understanding of the diversity of intervention methods employed by local governments in order to better engage with the complex dynamics of research use.

## Supplementary Information

**Additional file 1**: PRISMA Checklist for Reporting Systematic Reviews.

**Additional file 2**: Classification of public health: top two levels of all classes.

**Additional file 3**: Inclusion and Exclusion Criteria.

**Additional file 4**: Quality Appraisal.

**Additional file 5**: Study Details.

## Data Availability

The datasets used and/or analysed during the current study are available on Open Science Framework https://osf.io/s38qf/.

## Abbreviations

NCD: Non-communicable disease
KT: Knowledge translation
MMAT: Mixed Methods Quality Appraisal Tool
UK: United Kingdom
