# Supplementary material for: Research evidence use in local government-led public health interventions: a systematic review
Source: Health Res Policy Syst. 2023 Jul 3;21:67. doi: 10.1186/s12961-023-01009-2 (PMC10318787; doi:10.1186/s12961-023-01009-2)
Supplement: Supplementary file 2 — Additional file 2: Classification of public health: top two levels of all classes. [file 12961_2023_1009_MOESM2_ESM.docx]

**Classification of public health: top levels of all classes**

| **Top-level class** | **Level 2 subclasses** | | | |
| --- | --- | --- | --- | --- |
| Functions | Assess health of populations  Protect from threats to health | | Promote health & prevent disease, disability & injury | |
| *Primary:* |  |  |  |  |
| *Instrumental:* | Ensure public health capability | | Build the evidence base for public health | |
| Health issues | Health and well-being | | Injury | |
|  | Diseases and conditions | | Disability and functioning | |
| Determinants of health | Environmental | Socioeconomic | | Health system |
|  | Person-level | External causes of injury | |  |
| Methods | Advocacy & lobbying | Legislation & regulation | | Screening to detect disease/risk factors |
|  | Communicable disease control specific | Lifestyle advice | |  |
|  |  | Management of biological risk | | Social action |
|  | Community action |  |  | Social marketing |
|  | Community development | Monitoring & surveillance | | Training & workforce  development methods |
|  | Counselling |  |  |  |
|  | Diagnosis | Personal skills development | | Treatment methods |
|  | Directed investment |  |  | Urban planning methods |
|  | Environmental monitoring | Political action | | Vector control methods |
|  | Epidemiologic methods | Public policy development | | Waste management methods |
|  | Exercise of capabilities |  |  |  |
|  | Food safety methods | Radiation safety methods | | Other methods of intervention |
|  | Health education | Remediation of environment methods | |  |
|  | Health impact assessment |  |  |  |
|  | Immunisation | Research & evaluation | |  |
|  | Infection control | Road safety methods | |  |
| Settings | Educational settings | Home settings | | Includes LOCATIONS – classification of geographical areas (e.g. postcodes) |
|  | Healthcare settings | Workplace settings | |  |
|  | Local government & community settings | Transport settings | |  |
|  |  | Other settings | |  |
| Resources & infrastructure | Administrative infrastructure | Partnerships | | Time |
|  |  | Physical infrastructure | | Workforce |
|  | Information systems | Policies | | Workforce development capacity |
|  | Legislative infrastructure | Technical infrastructure | |  |
|  | Organisational systems |  | |  |

Source: Gruszin S, Jorm L, Churches T, Straton Su Gruszin J. Public Health Classifications Project Phase One: Final Report. Melbourne; 2006.
