# Supplementary material for: Research evidence use in local government-led public health interventions: a systematic review
Source: Health Res Policy Syst. 2023 Jul 3;21:67. doi: 10.1186/s12961-023-01009-2 (PMC10318787; doi:10.1186/s12961-023-01009-2)
Supplement: Supplementary file 3 — Additional file 3: Inclusion and Exclusion Criteria. [file 12961_2023_1009_MOESM3_ESM.docx]

**Inclusion and Exclusion Criteria**

| **Inclusion** | **Exclusion** |
| --- | --- |
| Primary quantitative and qualitative studies (including observational studies) published in peer-reviewed journal publications | Systematic reviews  Articles published in non-peer reviewed publications |
| English language | Non-English language |
| Published between 2000 - 2020 | Published prior to 2000 |
| *Study setting* | |
| Studies that report a public health intervention implemented within a local government setting *including:*  Studies that report an intervention implemented by a non-public health department (e.g. sustainability, planning) provided the intervention has an explicitly stated goal of improving population health outcomes | Studies that report a public health intervention implemented at the state or federal government level  Studies that report a public health intervention implemented by public health teams not embedded in a policy context (e.g. local health department, community health centre) |
| *Intervention focus & research use* | |
| Studies that report the use of research evidence in an intervention that directly targets population health outcomes *including:*  Studies that draw on research sourced from grey literature (e.g. policy briefs, agency reports or guidelines) | Studies reporting interventions that target individual health outcomes  Studies reporting knowledge translation interventions with the primary aim of increasing evidence use  Studies that do not report the use of research evidence |
| *Implementation* | |
| Studies that report a public health intervention initiated/led by local government decision-makers | Studies that report a public health intervention initiated/led by stakeholders that are external to local government such as state or federal government or universities |
